# Association of post-tonsillectomy fever with postoperative complications

**DOI:** 10.1007/s00405-026-10094-2

**Published:** 2026-03-05

**Authors:** Noa Sheffer, Itai Pansky, Itai hazan, Oded cohen, Inbal Golan- Tripto, Daniel Yafit, Nir Amitai, Oren Ziv

**Affiliations:** 1 Department of Otolaryngology-Head & Neck Surgery, Soroka University Medical Center, Faculty of Health Sciences, Ben-Gurion University of the Negev, Beer-Sheva, Israel; 2 Department of Otolaryngology-Head & Neck Surgery, Assuta Ashdod Hospital, Faculty of Health Sciences, Ben-Gurion University of the Negev, Beer Sheva, Israel; 3 Clinical Research Center, Soroka University Medical Center and, Faculty of Health Sciences, Ben-Gurion University of the Negev, Beer-Sheva, Israel; 4 Pediatric Pulmonary Unit, Saban Children’s Hospital, Soroka University Medical Center, Faculty of Health Sciences, Ben-Gurion University of the Negev, Beer Sheva, Israel

**Keywords:** Tonsillectomy, Adenoidectomy, Fever, Bleeding, Complications

## Abstract

**Objective:**

To investigate the incidence and risk factors associated with postoperative fever following tonsillectomy or adenotonsillectomy in pediatric patients, and explore its potential correlation with subsequent hemorrhage, emergency room (ER) visits, and rehospitalizations.

**Study design:**

Retrospective cohort study.

**Setting:**

Pediatric patients undergoing total or partial tonsillectomy or adenotonsillectomy between 2015 and 2022 across six medical centers.

**Methods:**

Patients were divided based on recorded postoperative temperature (within 24 h) into study (temperature ≥ 38 °C) and control group (Temperature < 38 °C). Primary outcomes included hemorrhage complications within 14 days post-procedure, secondary outcomes included ER visits and rehospitalizations.

**Results:**

This study included 2,512 pediatric patients; 426 in the study group and 2,086 in the control group. The study group exhibited significantly higher proportions of post-operative hemorrhages (6.8% vs. 3.4%, p < 0.001), increased ER visits (18% vs 9.6%, p < 0.001), elevated readmission rates (12% vs 5.7%, p < 0.001), and longer postoperative hospital stay (median 2.00 day [IQR 1.00, 2.00] vs 1.00 day [IQR 0.00, 1.00], p < 0.001) compared to the control group. Multivariate analyses controlled for age, sex, and operation type showed post-operative fever correlated significantly with hemorrhage, readmissions, and ER visits (OR 3.23, p < 0.001, OR 2.93, p < 0.001, OR 2.36, p < 0.001 respectively).

**Conclusions:**

Postoperative fever independently correlates with an increased risk of hemorrhage and readmission following tonsillectomy or adenotonsillectomy. These findings suggest that fever may serve as an early marker for complications, reinforcing its clinical importance in guiding postoperative care strategies and caregiver education.

**Level of evidence:**

3.

## Introduction

Tonsillectomy is a common pediatric surgical procedure in the United States, with a prevalence of 87.2 cases per 10,000 children under the age of 15. [1, 2] One of its significant postoperative complications is hemorrhage, [3] which can be life-threatening and is a leading cause for readmission [4–6], posing challenges for both families and hospitals. Around 2.9% of cases experience severe complications requiring hospital readmission [7]. It is crucial to address the issue of readmission, as it inconveniences families significantly and increases treatment costs for hospitals.

Many attempts have been made over the years to identify risk factors for this severe complication. [3–9] including the CHEER research that aimed to compare post-operative bleeding rates across different age groups, diagnoses, and practice types [3] and Gonçalves et al. who examined 897 patients undergoing tonsillectomy to assess postoperative hemorrhage incidence and associated risk factors [7].

Post-surgical fever following adenotonsillectomy is a relatively common phenomenon, which is usually regarded as viral infection or anesthesiology-related fever, and therefore not regarded as a complication of surgery. Furthermore, knowledge on the impact of post-adenotonsillectomy fever on surgical complications and course is lacking, with no significant research examining the question in the past 30 years [10], though the surgical technique has been revolutionized by the introduction of power intracapsular tonsil adenoidectomy (PITA) [11]. While the etiology of post-tonsillectomy fever is not fully understood, it is thought to be mainly associated with inflammatory reactions to tissue damage [10]. Our hypothesis suggests that fever may be a marker of the inflammatory process and could serve as an early sign of hemorrhage or other post-operative complication.

Therefore, the purpose of this study is to evaluate the impact of post adenotonsillectomy fever on hemorrhage, ER visits and rehospitalization. To the best of our knowledge, this is the largest study to describe the relation between postoperative fever and post-surgical complications conducted in the past 30 years [10].

## Methods

The study was approved by the institutional Helsinki committee (SOR 0265–22). This is a retrospective cohort study that includes all patients under 18 who underwent total or partial elective tonsillectomy or adenotonsillectomy between 2015 and 2022. The Health Maintenance Organization encompasses 6 different medical centers in Israel, including over 4.8 million patients [12, 13].

None of the patients had an active infection or received antibiotics within two weeks prior to the procedure. During the surgical procedure (tonsillectomy or adenotonsillectomy), all patients received corticosteroids and antipyretics at the time of anesthesia, with additional doses given in recovery room if necessary. Our study focused on the use of antipyretics after recovery, during hospitalization, and included all pediatric patients who underwent total or partial tonsillectomy or adenotonsillectomy (ICD9 codes- Z282, Z283, respectively (. Exclusion criteria included the following: (1) temperature was not measured during hospitalization; (2) patients who were treated with antipyretic medication during the first 24 h, potentially preventing fever, and (3) patients with insufficient data. The study was approved by the National Data Committee Review Board. The study group included all patients who underwent surgery and had at least one measurement of 38 °C within 24 h of the procedure. This group was compared to a control group, which included all patients undergoing surgery without a measured fever above 38 °C within 24 h after the operation while still in hospital. The adjusted models were controlled for age, sex and operation type.

The primary outcome is hemorrhage occurring within 14 days after the initial procedure. The secondary outcomes are ER visits and rehospitalizations within 14 days following the procedure.

The data was retrieved from Clalit health service’s computerized medical records using MDclone system. Retrieved data included age, sex, type of surgery (tonsillectomy or adenotonsillectomy), maximal measured temperature at the facility of operation at the first 24 h post procedure (Celsius), duration of hospitalization (days), surgical complications (bleeding and pain according to ICD9 9981, 33,818/33819 respectively), post-surgery complications during hospitalization (hemorrhage and acute pain according to ICD9 998.11, 338.1 respectively), visits to the ED in any indication within 14 days post-surgery, rehospitalization, and re-surgery due to hemorrhage after tonsillectomy (according to ICD9 Z218).

## Results

**Fig. 1:**
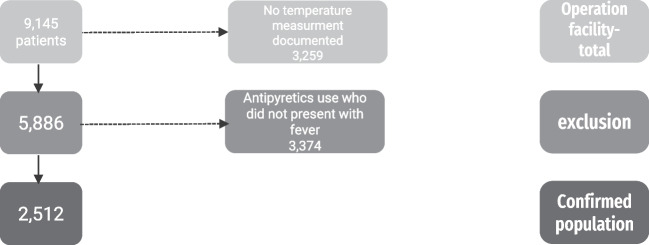
A flow chart showing exclusion criteria and the confirmed eligible population

Patient selection eligibility criteria are presented in Fig. 1. A total of 9,145 individuals were initially assessed for eligibility. After exclusion, the confirmed eligible population for the study is 2,512 patients; 426 in the study group and 2,086 as the control group.

Patients’ characteristics and surgery type are presented in Table 1. The study group was significantly younger than the control group (3.4 ± 1.9 vs. 5.7 ± 3.4 years, p < 0.001, respectively). The study group had a significantly higher proportion of adenotonsillectomy compared to control (97.7% vs. 94%, p < 0.001, respectively).

**Table 1:** Patient characteristics

Characteristic %(N)	Overall (N = 2,512)	Control Group (N = 2,086^1^)	Study Group (N = 426^1^)	p-value
Age (Years)	5.3 + −3.3	5.7 + −3.4	3.4 + −1.9	< 0.001
Sex (Male)	59 (1,475)	58 (1,203)	64 (272)	0.018
Operation				< 0.001
Adenotonsillectomy	95 (2,377)	94 (1,961)	98 (416)	
Tonsillectomy	5.4 (135)	6 (125)	2.3 (10)	
Duration of Hospitalization (Days)	2.00 (2.00, 2.00)	2.00 (1.00, 2.00)	3.00 (2.00, 4.00)	< 0.001
Antipyretics	16 (397)	0 (0)	93 (397)	< 0.001

^1^–Mean +—SD; % (n); Median (IQR)

^1^Wilcoxon rank sum test; Pearson’s Chi-squared test

The median time to fever onset was 3.8 h post-surgery (IQR: 1.7–8.2 h), with 86.6% of cases occurring within the first 12 h.

Comparisons of study outcomes are presented in Table 2. When comparing the study group to the control group, the study group had significantly higher proportions of post-operative hemorrhages (6.8% vs. 3.4%, p < 0.001, respectively); higher proportions of ER visits (18% vs 9.6%, p < 0.001, respectively); higher readmissions rates (12% vs 5.7%, p < 0.001, respectively), and longer postoperative hospital stay (2.00 day [median], IQR 1.00, 2.00 vs 1.00, IQR 0.00, 1.00, p < 0.001, respectively).

**Table 2:** Post-surgery evaluation and outcomes

Characteristic %(N)	Overall (N = 2,512)	Control Group (N = 2,086^1^)	Study Group (N = 426^1^)	p-value^2^
Hemorrhage	4 (100)	3.4 (71)	6.8 (29)	< 0.001
Acute pain	0.2 (6)	0.2 (4)	0.5 (2)	0.3
Post-Operative Hospital stay (Days)	1.00 (1.00, 1.00)	1.00 (0.00, 1.00)	2.00 (1.00,2.00)	< 0.001
ER visits	11 (277)	9.6 (200)	18 (77)	< 0.001
Readmission	6.8 (172)	5.7 (119)	12 (53)	< 0.001

_1_% (n); Median (IQR)

^2^ Pearson’s Chi-squared test; Fisher’s exact test; Wilcoxon rank sum test

Multivariable analyses are presented in Tables 3 and 4. Postoperative fever was significantly associated with postoperative hemorrhage (OR 3.23, p < 0.001, Table 3), readmissions, and ER visits (OR 2.93, p < 0.001, OR 2.36, p < 0.001 respectively, Table 4). In a sensitivity analysis excluding hospitals with fewer than 200 cases, the findings remained robust, with no change in the overall trend (OR = 3.27, 95% CI: 1.82–5.69).

**Table 3:** Association between different exposures and post-operative Hemorrhage

	Non adjusted model			Adjusted model		
**characteristic**	OR^1^	**95% **CI^1^	p-value	OR^1^	95% CI^1^	p-value
Fever	2.07	1.31,3.20	** < 0.001**	3.23	1.96,5.22	** < 0.001**
Age (Years)	1.12	1.07,1.18	** < 0.001**			
Sex (Male)	0.82	0.55,1.23	0.3			
Duration of Hospitalisation (Days)	1.02	0.90,1.08	0.6			
Operation						
Adenotonsillectomy	–	–				
Tonsillectomy	2.54	1.29, 4.59	**0.004**			

^1^OR = Odds Ratio, CI = Confidence Interval. The adjusted model controlled for age, sex and operation type

**Table 4:** Association between different exposures to post operative readmissions and ER visits

	post-operative Readmission						post operative ER visits					
	Non adjusted model			Adjusted model			Non adjusted model			Adjusted model		
characteristic	OR^1^	95% CI^1^	p-value	OR^1^	95% CI^1^	p-value	OR^1^	95% CI^1^	p-value	OR^1^	95% CI^1^	p-value
Fever	2.35	1.66, 3.29	** < 0.001**	2.93	2.02, 4.22	** < 0.001**	2.08	1.56, 2.76	** < 0.001**	2.36	1.73, 3.18	** < 0.001**
Age (Years)	1.05	1.01, 1.10	0.021				1.02	0.98, 1.06	0.2			
Sex (Male)	0.97	0.71, 1.34	0.9				0.93	0.72, 1.19	0.5			
Duration of Hospitalisation (Days)	1.01	0.90, 1.07	0.8				0.97	0.86, 1.04	0.5			
Operation												
Adenotonsillectomy	–	–					–	–				
Tonsillectomy	2.07	1.17, 3.43	**0.008**				1.43	0.85, 2.30	0.2			

^1^OR = Odds Ratio, CI = Confidence Interval. The adjusted models controlled for age, sex and operation type

## Discussion

The main goal of the study was to assess the association between post tonsillectomy or adenotonsillectomy fever 24 h after surgery, and post-surgical complications- hemorrhage, ER visits, and readmission. After logistic regression, our study shows a significant association between fever and the aforementioned [8, 9, 12–15]. Many studies have addressed the issue of complications, but most have not considered fever as a risk factor [3–8, 14]. Granell et al. conducted a six-year retrospective review to assess the safety of ambulatory tonsillectomy and examined postoperative complications, but did not evaluate the relationship between fever and these complications [15].

Our logistic regression analysis has shown that postoperative fever is significantly associated with hemorrhage, leading us to explore the underlying reasons for this association. While it is common to experience self-limited "physiological" fever after most major surgeries, the etiology of post-surgical fever is not fully understood [16]. Past studies have associated it with inflammatory reactions to tissue damage, anesthetic agents, and bacteremia [17]. However, other studies have failed to demonstrate a significant correlation between positive blood cultures and postoperative fever [10]. This suggests that early onset post-tonsillectomy fever (within 24 h) is more plausibly explained by a physiological inflammatory response rather than infectious etiology. This is particularly relevant in pediatric tonsillectomy, where tissue trauma, thermal injury, and healing dynamics are rapid.

Early fever may indicate an increased inflammatory reaction in the tonsillar bed. During acute inflammatory responses, leukocyte-derived mediators like histamine and bradykinin, along with vasodilatory mediators such as prostaglandins, cause arteriolar dilation and increased blood flow in response to proinflammatory molecules. Additionally, complement components like C5a induce various types of acute inflammation, including vasodilation and locally increased blood flow. Inflammation also leads to a reduction in platelet activity as platelets adhere to white blood cells at sites of inflammation, forming aggregates and decreasing the number of circulating thrombocytes. C-reactive protein (CRP) amplifies this process by promoting platelet phagocytosis. These factors collectively explain the association between postoperative fever and hemorrhage [18–20].

Several studies have attempted to stratify populations at risk for postoperative hemorrhage [8, 9, 21, 22], and recent findings support our assumptions regarding the link between inflammation and hemorrhage. Martin et al. found that perioperative COVID-19 infection increases the risk of postoperative bleeding, highlighting the possible link between inflammation caused by COVID-19 and postsurgical hemorrhage [23]. Furthermore, Kang et al. found that in children undergoing adenotonsillectomy, dexamethasone did not raise the risk of postoperative hemorrhage [24]. This finding is significant as it shows that corticosteroids, which are known to reduce the synthesis of prostaglandins, did not increase hemorrhage risk, supporting the idea that controlled inflammation does not exacerbate bleeding, whereas unchecked inflammation might.

Some researchers have investigated the risk factors for higher post-surgical readmission rates, which, in turn, cause significant inconvenience to families and substantially increases treatment costs for hospitals. [25] an issue we address in our research. Johnson et al. explored the risk factors and causes associated with hospital readmission after pediatric tonsillectomy and adenotonsillectomy. They identified several risk factors, with the leading causes being hemorrhage, dehydration, and pain, but did not address fever [14]. Similarly, Alsalamah et al. researched the predictors of readmission following tonsillectomy in the pediatric population and found that hemorrhage and dehydration were the leading causes of readmission. They noted that fever was less frequently reported as a cause of readmission in their study, in contrast to our results, which show a significant correlation between fever and readmission [26].

Our study has some limitations. Following surgery, many patients are prescribed antipyretics. These medications can impact body temperature and potentially obscure the presence of fever, affecting emergency room visits and post-surgery complications. To mitigate the potential influence of antipyretics on result accuracy, patients who were treated with antipyretics and did not present with fever were exclude. Patients who were treated with antipyretics and did present with fever were considered “exposed”, and were thus included in the study group. Another limitation could be the diverse approaches and experience levels among surgeons conducting the surgeries, which introduce inter-surgeon variability. These differences in surgeon factors could potentially confound the results. However, the substantial sample size, including both patients and surgeons, helped minimize the influence of inter-surgeon variability. Also, Anand et al. found that there was no relation between surgeon experience and appearance of fever, [10] and Rajpal et al. found there was no correlation between surgeon experience and length of hospitalization [27]. Additionally, variations in surgical procedures (complete vs partial tonsillectomy) may influence complications. Since partial and total tonsillectomy are both coded with the same ICD- 9 code, we were unable to differentiate the two retrospectively. To address this, we examined the confounding effect of these variations through a multivariable analysis, which included the reason for the surgery and the patient’s age.

Due to the limited sample size and the relatively low incidence of complications, survival analysis could not be performed in this study. Future research with larger multicenter or international cohorts and extended follow-up would allow for such analyses and provide more comprehensive insights into long-term outcomes.

## Conclusion

Post-operative fever independently correlates with increased risk for postoperative hemorrhage and readmissions, suggesting its potential as an early marker for complications. Clinicians should take these findings into account when deciding on the appropriate length of stay following surgery, as well as targeted parental education in ‘at risk’ populations.
